# Processing of Nuclear Viroids In Vivo: An Interplay between RNA Conformations

**DOI:** 10.1371/journal.ppat.0030182

**Published:** 2007-11-30

**Authors:** María-Eugenia Gas, Carmen Hernández, Ricardo Flores, José-Antonio Daròs

**Affiliations:** Instituto de Biología Molecular y Celular de Plantas, CSIC-Universidad Politécnica de Valencia, Valencia, Spain; The Scripps Research Institute, United States of America

## Abstract

Replication of viroids, small non-protein-coding plant pathogenic RNAs, entails reiterative transcription of their incoming single-stranded circular genomes, to which the (+) polarity is arbitrarily assigned, cleavage of the oligomeric strands of one or both polarities to unit-length, and ligation to circular RNAs. While cleavage in chloroplastic viroids (family Avsunviroidae) is mediated by hammerhead ribozymes, where and how cleavage of oligomeric (+) RNAs of nuclear viroids (family Pospiviroidae) occurs in vivo remains controversial. Previous in vitro data indicated that a hairpin capped by a GAAA tetraloop is the RNA motif directing cleavage and a loop E motif ligation. Here we have re-examined this question in vivo*,* taking advantage of earlier findings showing that dimeric viroid (+) RNAs of the family Pospiviroidae transgenically expressed in *Arabidopsis thaliana* are processed correctly. Using this methodology, we have mapped the processing site of three members of this family at equivalent positions of the hairpin I/double-stranded structure that the upper strand and flanking nucleotides of the central conserved region (CCR) can form. More specifically, from the effects of 16 mutations on *Citrus exocortis viroid* expressed transgenically in A. thaliana, we conclude that the substrate for in vivo cleavage is the conserved double-stranded structure, with hairpin I potentially facilitating the adoption of this structure*,* whereas ligation is determined by loop E and flanking nucleotides of the two CCR strands. These results have deep implications on the underlying mechanism of both processing reactions, which are most likely catalyzed by enzymes different from those generally assumed: cleavage by a member of the RNase III family, and ligation by an RNA ligase distinct from the only one characterized so far in plants, thus predicting the existence of at least a second plant RNA ligase.

## Author Summary

Interactions of viroids with their host plants are unique because these subviral pathogenic RNAs lack protein-coding capacity. Therefore, hosts must provide all enzymes and auxiliary factors that viroids need for their infectious cycle. Replication of viroids entails reiterative transcription of their single-stranded circular genomes, cleavage of oligomeric strands to unit-length, and ligation to circular RNAs. While cleavage in chloroplastic viroids (family Avsunviroidae) is autocatalytic and mediated by hammerhead ribozymes, where and how cleavage of oligomeric (+) RNAs of nuclear viroids (family Pospiviroidae) occurs in vivo is controversial. We have re-examined this question in vivo, taking advantage that dimeric viroid RNAs expressed transgenically in Arabidopsis thaliana are processed correctly. Together with mapping the in vivo processing site of three members of the family Pospiviroidae, our results with 16 mutants of one of these viroids support that cleavage is directed by an RNA motif conserved in all members of the family, and ligation by an extended conformation containing a motif termed loop E. Both processing reactions are most likely catalyzed by enzymes different from those generally assumed: cleavage by an RNase III–like enzyme, and ligation by an RNA ligase distinct from the only one characterized so far in plants.

## Introduction

Viroids, plant pathogens with a minimal non-protein-coding circular RNA genome of 246–401 nt [1], are classified into two families. The members of the first, Pospiviroidae, replicate in the nucleus through an asymmetric rolling-circle mechanism, have a central conserved region (CCR), and cannot form hammerhead ribozymes. The members of the second, Avsunviroidae, replicate in the chloroplast through a symmetric rolling-circle mechanism, lack a CCR, and can form hammerhead ribozymes [2–4]. In *Potato spindle tuber viroid* (PSTVd) [5,6], the type species of the genus *Pospiviroid* (family Pospiviroidae), the incoming monomeric circular RNA, to which (+) polarity is arbitrarily assigned, is reiteratively transcribed by the nuclear RNA polymerase II into oligomeric (−) RNAs that in turn serve as template for synthesis of oligomeric (+) RNAs. These latter transcripts are then cleaved and ligated to the mature viroid circular RNA [7,8]. In *Avocado sunblotch viroid* (ASBVd) [9], the type species of the family Avsunviroidae, the oligomeric (−) RNAs generated by a chloroplastic RNA polymerase are cleaved and ligated before serving as template for a second rolling-circle leading to the mature viroid circular RNA. In this family, the oligomeric RNA intermediates of both polarities self-cleave through hammerhead ribozymes [10,11]. In contrast, cleavage and ligation of oligomeric (+) RNAs in the family Pospiviroidae is catalyzed by host enzymes [12–14], which recognize particular RNA motifs.

Early infectivity bioassays with viroid RNAs containing repeated sequences of the upper CCR strand [15–18] implicated these sequences in processing of the oligomeric (+) strands of the family Pospiviroidae through the adoption of either hairpin I, a metastable motif that can be formed by the upper CCR strand and flanking nucleotides during thermal denaturation [19], or through a thermodynamically stable double-stranded structure that can be alternatively assumed by the same sequences of a dimeric (or oligomeric) RNA [15,18] (Figure S1). More recently, in vitro and thermodynamic analyses of the products obtained by incubating a potato nuclear extract with a full-length PSTVd RNA containing a 17-nt repeat of the upper CCR strand have led to the proposal that cleavage of (+) strands is driven by a multibranched structure with a hairpin—different from hairpin I—capped by a GAAA tetraloop conserved in members of the genus *Pospiviroid*, which subsequently switches to an extended conformation with a loop E that promotes ligation [20] (Figure S1). Similar results have been obtained with a reduced version of this construction containing the GAAA tetraloop and loop E [21]. Loop E, a UV-sensitive motif of RNA tertiary structure that is conserved in PSTVd and members of its genus [3] and exists in vitro [22] and in vivo [23,24], has been also involved in host specificity [25], pathogenesis [26], and transcription [27]. The structural model based on isostericity matrix and mutagenic analyses derived recently for PSTVd loop E [27] can be extended to CEVd. However, the proposed cleavage-ligation mechanism [20] may not apply to other members of the family Pospiviroidae that cannot form the GAAA tetraloop and loop E [3]. Moreover, alternative processing sites in the lower CCR strand, or outside this region, have been observed for several members of the family Pospiviroidae [28–32], with a proposal even suggesting that cleavage could be autocatalytic, albeit mediated by non-hammerhead ribozymes [33].

Here we have re-examined this question in vivo using a system based in transgenic Arabidopsis thaliana expressing dimeric (+) transcripts of *Citrus exocortis viroid* (CEVd), *Hop stunt viroid* (HSVd), and *Apple scar skin viroid* (ASSVd) [34] of the genera *Pospiviroid*, *Hostuviroid*, and *Apscaviroid*, respectively, within the family Pospiviroidae [3]. In addition to mapping what we believe is their major processing site in vivo*,* data obtained with 16 CEVd mutants support a previous model involving the hairpin I/double-stranded structure formed by the upper CCR strand and flanking nucleotides in cleavage [15], and loop E and flanking nucleotides of both strands in ligation. A corollary of our results is that the RNase and RNA ligase that catalize both processing reactions are most likely different from those generally assumed so far.

## Results

### Correct Processing of Dimeric CEVd (+) Transcripts in A. thaliana

**Figure 1:**
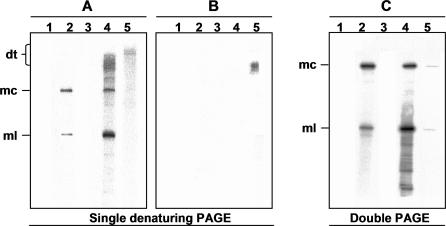
Northern Blot Analysis of CEVd RNAs in Gynura and A. thaliana RNAs were separated by single denaturing PAGE (A and B) or double PAGE (C) and hybridized with a probe for detecting CEVd (+) strands (A and C) or CEVd (−) strands (B). Lanes 1, non-inoculated gynura. Lanes 2, CEVd-infected gynura. Lanes 3, non-transgenic A. thaliana. Lanes 4 and 5, transgenic A. thaliana expressing dt CEVd (+) and (−) RNAs, respectively. Lanes 5 were loaded with twice the RNA amount applied to lanes 4. Positions of the dt, mc, and ml CEVd RNAs are indicated.

We first re-examined where the processing site occurs in vivo*.* This question could be tackled by mapping the 5′ termini of the monomeric linear (ml) viroid (+) strands isolated from infected propagation hosts (e.g., gynura for CEVd). However, the contribution of nicked byproducts of the monomeric circular (mc) viroid (+) RNA—the most abundant replication product—resulting from in vivo turnover or artifactual degradation during purification has precluded an unambiguous analysis so far. As an alternative, we evaluated the A. thaliana–based transgenic system established recently [34]. More specifically, an *A. thaliana* transgenic line expressing a CEVd (+) dimeric transcript (dt) [34] was chosen. Northern blot hybridization of RNAs separated by denaturing PAGE showed that plants of this line accumulate, besides the dt viroid (+) RNAs, the mc and ml CEVd (+) forms; however, in contrast to the situation in CEVd-infected gynura, the ml RNA was more abundant than its circular counterpart (Figure 1A and 1C, compare lanes 2 and 4). This indicates that A. thaliana can process correctly the dt CEVd (+) RNAs, with cleavage being more efficient than circularization, thus reducing the contribution of nicked mc (+) RNAs to the population of ml (+) RNAs present in vivo*.* RT-PCR amplification, cloning, and sequencing of the mc CEVd (+) forms extracted from the transgenic A. thaliana line confirmed that they were full-length [34], and infectious when mechanically inoculated to tomato (unpublished data). Furthermore, northern blot hybridization of another transgenic A. thaliana line expressing a dt CEVd (−) RNA showed low accumulation levels of the mc (+) RNA (Figure 1C, lane 5), indicating that despite not being a typical host, *A. thaliana* has the enzymatic machinery for replicating CEVd, in line with previous results for HSVd [34].

### The Cleavage Site of Dimeric CEVd (+) Transcripts Maps at Specific Positions of the Hairpin I/Double-Stranded Structure Formed by the Upper CCR Strand

**Figure 2:**
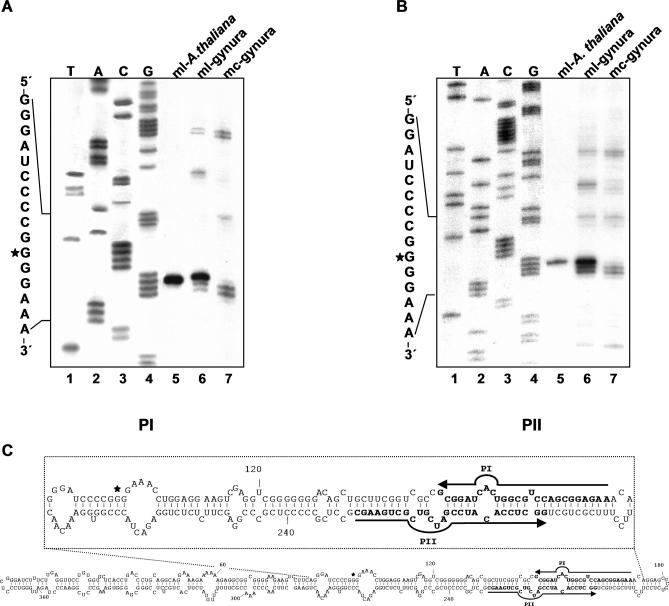
Primer Extensions on the Monomeric Circular and Linear CEVd RNAs (A and B) The cDNAs generated with the CEVd (−) primers PI (A) and PII (B) were separated by denaturing PAGE. Lanes 1 to 4, sequencing ladders obtained with PI and pBmCEVdB (A), and PII and pBmCEVdS (B). Lanes 5 and 6, cDNAs from reverse transcription of ml CEVd (+) RNAs purified from transgenic A. thaliana expressing a dt CEVd (+) RNA, and from CEVd-infected gynura, respectively. Lanes 7, cDNAs from reverse transcription of mc CEVd (+) RNA purified from CEVd-infected gynura. A portion of the CEVd (+) sequence is indicated on the left with asterisks marking the 5′-terminal nucleotide of the linear forms. (C) Rod-like secondary structure predicted for CEVd, with the upper inset highlighting a portion thereof. Positions of the complementary primers PI and PII are indicated with arrows and bold fonts, and the processing site with an asterisk.

**Figure 3:**
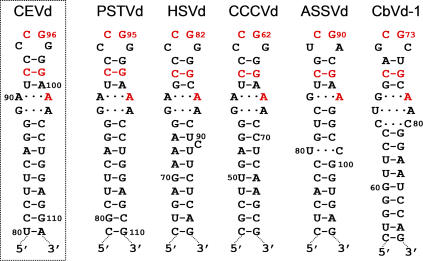
Hairpin I Structures of the Five Type Species of the Family Pospiviroidae This structural element is formed by the upper CCR strand and flanking nucleotides of PSTVd, HSVd, CCCVd (*Coconut cadang-cadang viroid*), ASSVd, and CbVd-1 (*Coleus blumei viroid 1*) [35]. Red fonts indicate conserved nucleotides in structurally similar positions. Continuous and broken lines represent Watson-Crick and non-canonical base pairs, respectively. Notice that the variability preserves the overall structure of hairpin I, including the terminal palindromic tetraloop, the adjacent 3-bp stem, and the long stem. Left inset, hairpin I of the wild-type CEVd variant used in the present work to transform A. thaliana (notice two co-variations with respect to PSTVd at the basis of the long stem).

RNAs from the transgenic A. thaliana line expressing the dt CEVd (+) species were separated by denaturing PAGE, and the positions in the gel of the mc and ml (+) CEVd RNA were inferred using a purified marker stained with ethidium bromide. RNAs migrating in the region of ml CEVd (+) RNA were eluted and examined by northern blot hybridization with a CEVd-specific probe that excluded the presence of other viroid RNA species (unpublished data). Preliminary length estimation of the CEVd-cDNAs from extensions on this RNA with 5′-end labeled primers PI, PIV, and PV, run in denaturing gels in parallel to RNA markers of known size, mapped the processing site around position 100 (Figure S2). Further analysis of the CEVd-cDNAs from extensions on the same RNA with the proximal 5′-end labeled primers PI and PII (Figure 2C), also run in denaturing gels but this time in parallel to sequencing ladders, revealed with both primers single bands corresponding to stops at position G97 (Figure 2A and 2B, lanes 5). These results identified the processing site between G96 and G97, which occupy the third and fourth positions of the tetraloop capping hairpin I (Figure 3), and two central positions of the double-stranded structure that the upper CCR strand and flanking nucleotides can form in di- or oligomeric viroid RNAs (see below). The secondary structure model here presented for hairpin I (Figure 3), with a capping tetraloop [18,35], differs from the original with a capping loop of 14 nt inferred from thermal denaturation studies with PSTVd [19]. As controls, ml and mc CEVd (+) RNAs obtained in parallel from CEVd-infected gynura were also analyzed. Prominent bands resulting from stops at G97 were also observed for the ml CEVd (+) RNA (Figure 2A and 2B, lanes 6), although accompanied by others (particularly in the extension with PII). Some of the extra bands were also observed for the mc CEVd (+) RNA, suggesting that they arise from elements of secondary structure, but others were not, thus supporting the contribution of nicked circular forms to the population of linear forms (Figure 2A and 2B, lanes 7). Altogether, these results identified in CEVd (+) strands a major processing site in vivo located in the upper CCR strand.

### The Cleavage Sites of Transgenic Dimeric (+) Transcripts from Other Members of the Family Pospiviroidae Also Map at Structurally Similar Positions

**Figure 4:**
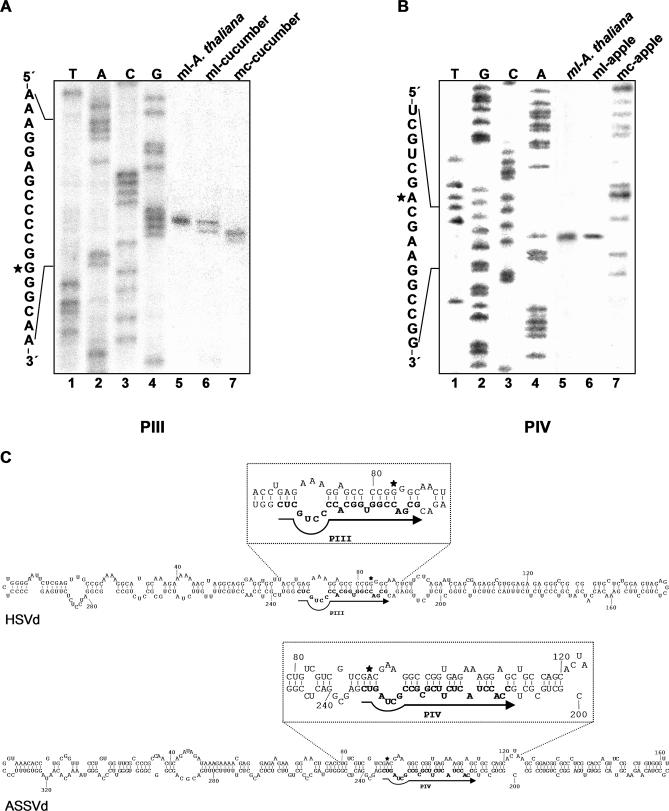
Primer Extensions on the Monomeric Circular and Linear HSVd and ASSVd RNAs (A and B) The cDNAs generated with the HSVd (−) primer PIII (A) and the ASSVd (−) primer PIV (B) were separated by denaturing PAGE. Lanes 1 to 4, sequencing ladders obtained with PIII and pBmHSVdE (A), and PIV and pBmASSVdS (B). Lanes 5, cDNAs from reverse transcription of ml HSVd (A) and ASSVd (B) (+) RNAs purified from transgenic A. thaliana expressing dt HSVd and ASSVd (+) RNAs, respectively. Lanes 6, cDNAs from reverse transcription of ml HSVd (A) and ASSVd (B) (+) RNAs purified from HSVd-infected cucumber and ASSVd-infected apple, respectively. Lanes 7, cDNAs from reverse transcription of mc HSVd (A) and ASSVd (B) (+) RNAs purified from HSVd-infected cucumber and ASSVd-infected apple, respectively. Portions of the HSVd and ASSVd (+) sequences are indicated on the left with asterisks marking the 5′-terminal nucleotide of the linear forms. (C) Rod-like secondary structure predicted for HSVd and ASSVd, with the upper insets highlighting a portion thereof. Positions of the complementary primers PIII and PIV are indicated with arrows and bold fonts, and the processing sites with asterisks.

To explore how general this finding was, processing was also studied in two additional members of the family Pospiviroidae, each with a characteristic hairpin I/double-stranded structure: HSVd and ASSVd [3,35]. RNA preparations containing the ml HSVd and ASSVd (+) RNAs as the only viroid species were isolated from two transgenic A. thaliana lines expressing the corresponding dt viroid (+) RNAs. However, in contrast to the situation observed in the CEVd-expressing line, these transgenic lines accumulated similar or more mc viroid (+) forms than their ml counterparts [34]. Parallel RNA preparations from HSVd-infected cucumber and ASSVd-infected apple, as well as the purified mc viroid (+) RNAs, were also analyzed. Extension with primer PIII identified the HSVd processing site at G82-G83 and extension with primer PIV identified the ASSVd processing site at G90-A91 (Figure 4). These two sites map, like in CEVd, between the third and fourth nucleotide of the tetraloop capping hairpin I (Figure 3), and at two central positions of the double-stranded structure formed by the upper CCR strand and flanking nucleotides in di- or oligomeric viroid RNAs (see below). It is pertinent in this context to note that the processing site here identified for ASSVd does not coincide with the corresponding site of *Citrus viroid-III* (also of the genus *Apscaviroid*) predicted from thermodynamic analysis and comparisons with PSTVd [36]. An alternative hairpin capped by a GAAA tetraloop, the RNA motif proposed to direct cleavage in a potato nuclear extract primed with a ml PSTVd (+) RNA containing a 17-nt repetition of the upper CCR strand [20], can neither be formed by HSVd nor by ASSVd*.* However, the PSTVd processing site inferred with this in vitro system [20] was in a position equivalent to that mapped here for CEVd with the A. thaliana–based in vivo system.

Collectively, these results strongly suggest a role for the hairpin I/double-stranded structure in directing cleavage in vivo of the oligomeric (+) RNAs in the family Pospiviroidae. If this double-stranded structure is indeed the substrate for the cleavage reaction, the enzyme involved would be most likely an RNase III. Intriguingly, the cleavage sites in each strand of the proposed substrate are separated by two 3′-protruding nucleotides, as also occurs in reactions catalyzed by enzymes of this class (see below).

### Effects of Mutations in the Central Positions of the Upper CCR Strand on Processing of Dimeric CEVd (+) Transcripts

**Figure 5:**
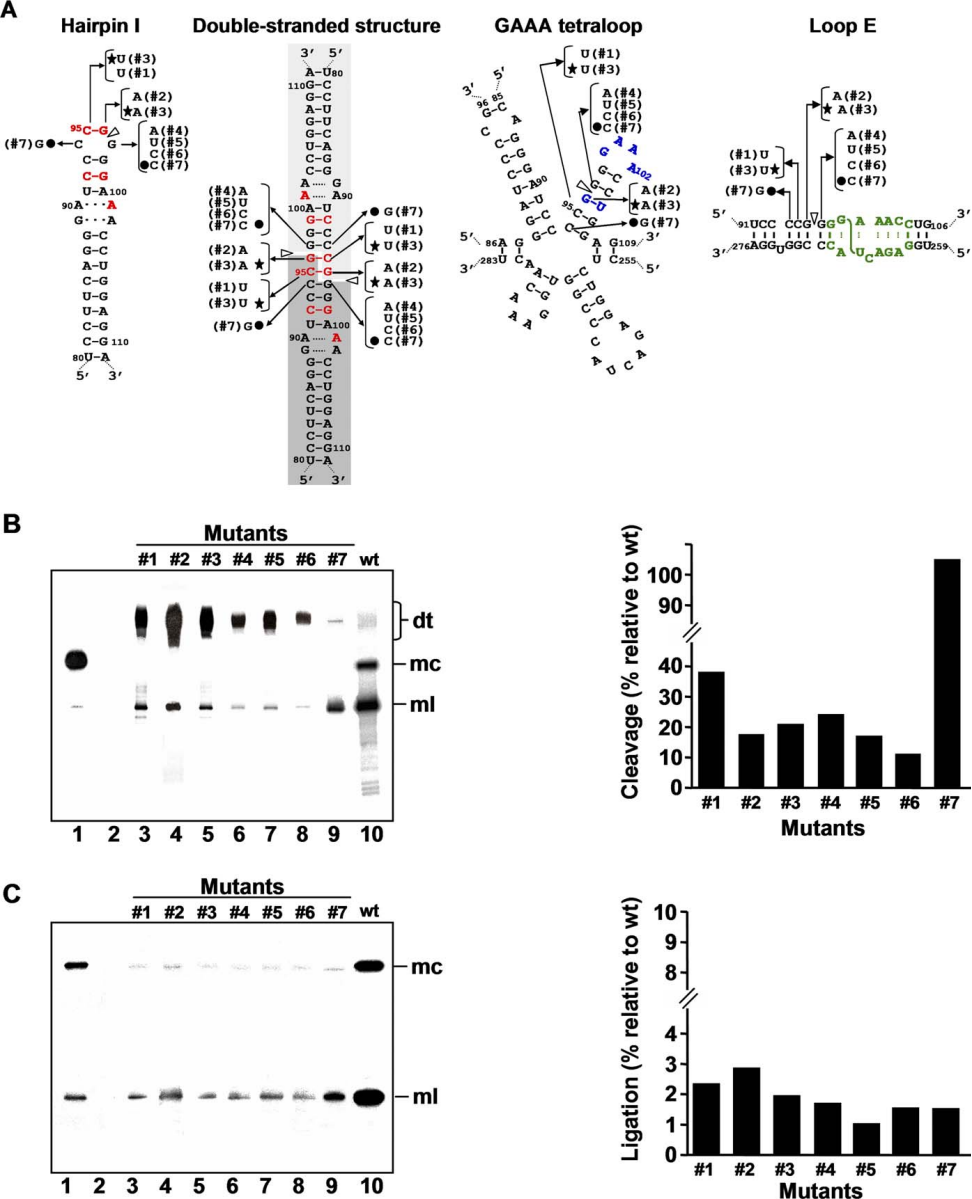
Processing in A. thaliana of Dimeric CEVd Transcripts with Mutations in the Central Positions of the Upper CCR Strand (A) Alternative foldings that can form the upper or both CCR strands: hairpin I/double-stranded structure in which red fonts denote nucleotides conserved in the family Pospiviroidae and light and dark gray backgrounds the two symmetric halves of the double-stranded structure (left), multibranched conformation with a GAAA-capped hairpin in which blue fonts denote conserved nucleotides in the genus *Pospiviroid* (center), and extended conformation containing a bulged-U helix adjacent to the loop E motif in which green fonts denote characteristic nucleotides by comparison with that of PSTVd [27] and the S-shaped line the UV-induced cross-link (right). White arrowheads mark the cleavage sites, and continuous and broken lines between nucleotides represent Watson-Crick and non-canonical base pairs, respectively. Nucleotide substitutions specific to each mutant (#1 to #7) are indicated on the alternative foldings, with filled circles and stars identifying the two double substitutions. (B) *Left panel.* Northern blot analysis of viroid-enriched RNAs separated by single denaturing PAGE, transferred to a membrane and hybridized with a radioactive riboprobe for detecting CEVd (+) strands. Lane 1, CEVd-infected gynura; lane 2, non-transformed *A. thaliana.* Lanes 3 to 9, transgenic A. thaliana expressing dt CEVd (+) RNAs with different mutations (#1 to #7, respectively). Lane 10, transgenic A. thaliana expressing dt CEVd (+) RNA wild-type (wt). Positions of dt, mc, and ml CEVd RNAs are indicated. *Right panel.* Histograms that represent cleavage of each mutant relative to the wt sequence. (C) *Left panel.* Northern blot analysis of viroid-enriched RNAs, the same as in panel (B) *left*, separated by double PAGE*. Right panel.* Histograms that represent ligation of each mutant relative to the wt sequence. Histogram values were estimated as detailed in Materials and Methods. Cleavage and ligation values of the wt sequence were 95% and 23%, respectively.

To gain further support for the RNA motif(s) directing processing in vivo, we determined how 16 different mutations (Table 1) affected cleavage and ligation of dt CEVd (+) RNAs expressed transgenically in *A. thaliana.* We selected the mutations according to their potential discriminatory effects on: i) the GAAA tetraloop [20], ii) the hairpin I/double-stranded structure formed by the upper CCR strand, and iii) the loop E motif formed by a subset of nucleotides of the upper and lower CCR strands (Figure 5A). It should be noticed that single mutations affect one position in the GAAA-capped hairpin and in hairpin I, but two positions in the double-stranded structure; similarly, the double mutations affect two positions of the hairpin structures, and four positions of the double-stranded structure. For an easier understanding we have clustered the mutations in three groups: those located in central positions of the upper CCR strand, in peripheral positions of the upper CCR strand, and in the lower CCR strand (the effects of the two latter groups will be presented in the two following sections). RNA preparations from the 16 transgenic lines, and from the line expressing wild-type (wt) CEVd, were analyzed by northern blot hybridization after single denaturing PAGE (in which the dt RNAs and their ml and mc processing products are separated) (Figure 5B), or double PAGE (in which better resolution of the ml and mc forms is achieved) (Figure 5C). Mutations in the viroid processing products were confirmed by cloning and sequencing the viroid circular RNA from different A. thaliana transgenic lines.

**Table 1:**
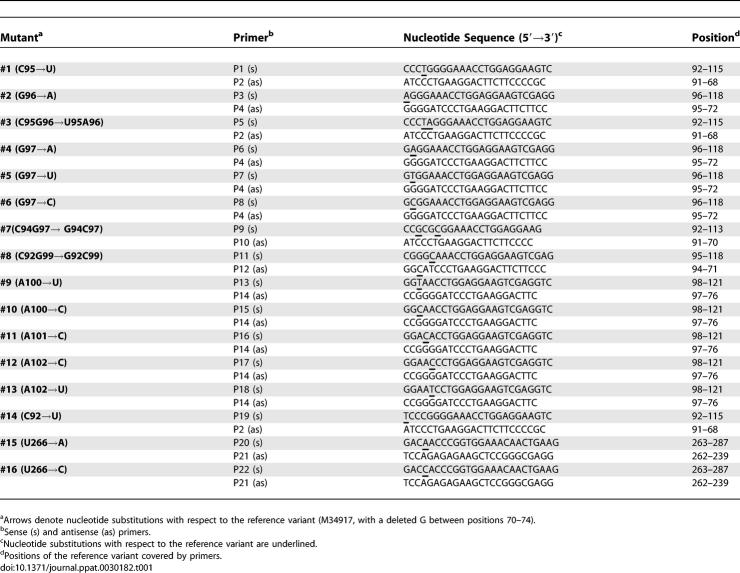
Primers for Site-Directed Mutagenesis of CEVd

Mutant #1 (C95→U) has no effect on the stem stability of hairpin I and only debilitates the stem of the GAAA-capped hairpin (a pair G:C is converted into G:U) (Figure 5A). However, in the double-stranded structure, this mutation affects a base pair phylogenetically conserved in the family Pospiviroidae (Figure 3) located in positions very close to the cleavage sites of both strands (Figure 5A). Therefore, if cleavage is directed by the double-stranded structure, changes in these positions are expected to have a negative influence; this was the case, with cleavage being reduced to 38% with respect to wt (Figure 5B).

Results with mutant #2 (G96→A) also support this view because the substitution has no effect on the stem stability of hairpin I and strengthens the stem stability of the GAAA-capped hairpin (a pair G:U is converted into A:U) (Figure 5A). But in the double-stranded structure this mutation affects a base pair also conserved in the family Pospiviroidae and adjacent in both strands to the cleavage sites, which are no longer embedded in an uninterrupted GC-rich helix (Figure 5A). Cleavage was reduced to less than 20% with respect to wt, consistent with a key role of the double-stranded structure in this reaction (Figure 5B).

The corresponding double mutant #3 (C95→U and G96→A) does not essentially alter the stem stability of both hairpin I and the GAAA-capped hairpin but, in contrast to the single mutant #2, restores the stability of the double-stranded structure (two contiguous G:C pairs are substituted by A:U pairs) (Figure 5A). However, cleavage was not restored (Figure 5B), indicating a requirement either for a particular sequence of the two nucleotides preceding the cleavage sites, or for a high thermodynamic stability of the secondary structure in the surrounding region (in which G:C pairs are prevalent).

Mutants #4 (G97→A), #5 (G97→U), and #6 (G97→C), have no influence on the stem stability of hairpin I or weaken the stem stability of the GAAA-capped hairpin (a C:G pair is disrupted). In the double-stranded structure mutations at this position affect nucleotides in both strands adjacent to both cleavage sites, which as in mutant #2 are no longer embedded in a double-stranded region (Figure 5A). Although reduction of cleavage (less than 25% with respect to wt, Figure 5B) supports also the involvement of the double-stranded structure in this reaction, these data can be alternatively interpreted as resulting from a destabilization of the GAAA-capped hairpin. However, cleavage was totally recovered in the double mutant #7 (G97→C and C94→G), in which the stability of the double-stranded structure was restored (these are indeed the nucleotides existing in the corresponding positions of CbVd-1, see Figure 3), whereas the GAAA-capped hairpin was further destabilized, thus providing additional credence to the role of the double-stranded structure in directing cleavage (Figure 5A and 5B).

The seven mutants of this group, despite not affecting nucleotides of loop E (Figure 5A), had a marked negative effect on ligation (Figure 5C). These results indicate that the sequence and/or secondary structure requirements for this reaction are more demanding than those regarding cleavage, and that they include nucleotides apart from those of loop E. The adjacent bulged-U helix (Figure 5A), the stability of which is affected by most of these mutants, appears particularly relevant in this respect.

### Effects of Mutations in Peripheral Positions of the Upper CCR Strand on Processing of Dimeric CEVd (+) Transcripts

**Figure 6:**
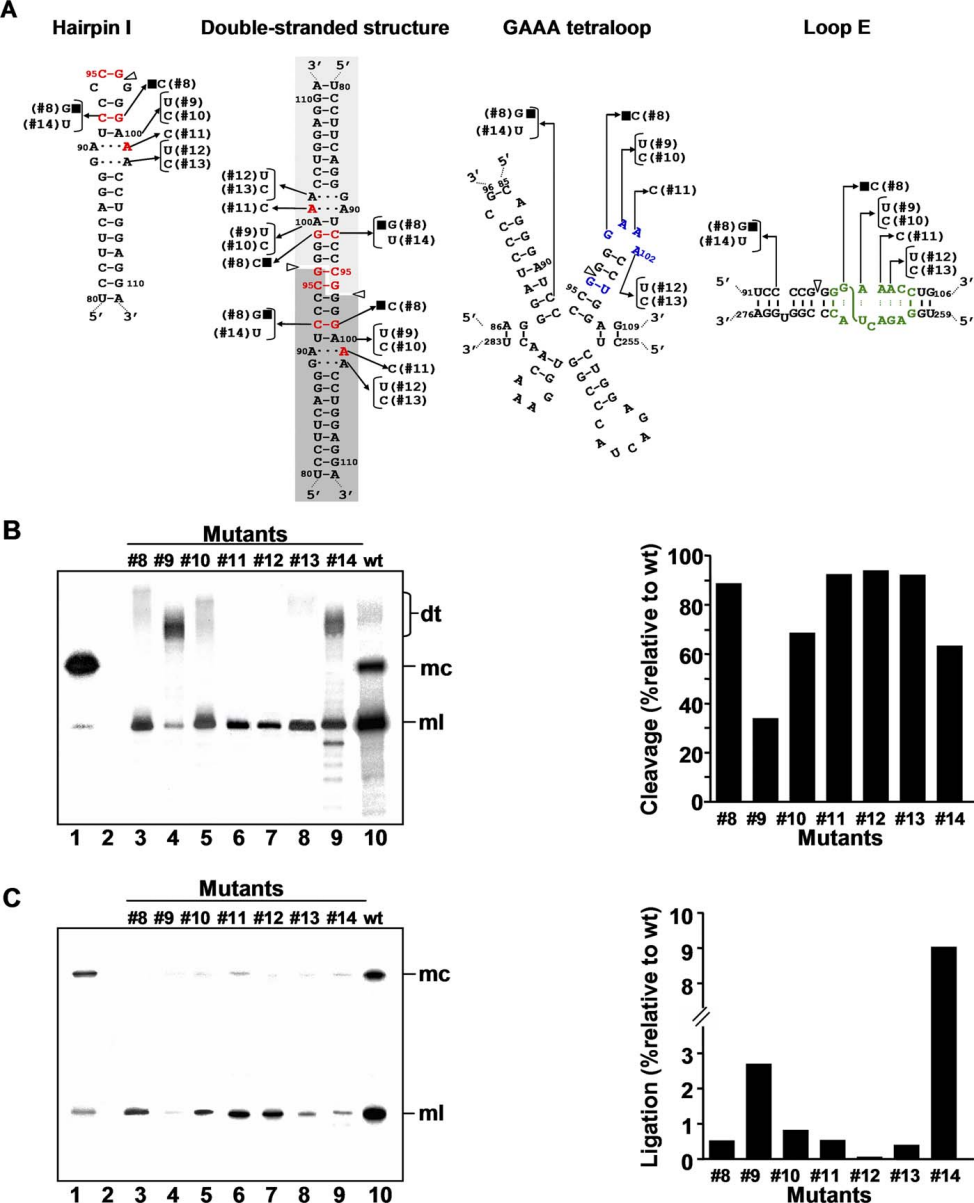
Processing in A. thaliana of Dimeric CEVd Transcripts with Mutations in Peripheral Positions of the Upper CCR Strand (A) Alternative foldings that can be formed by the upper or both CCR strands. Nucleotide substitutions specific of each mutant (#8 to #14) are indicated on the alternative foldings, with filled squares identifying the double substitution. (B) *Left panel.* Northern blot analysis of viroid-enriched RNAs separated by single denaturing PAGE, transferred to a membrane, and hybridized with a radioactive riboprobe for detecting CEVd (+) strands. Lane 1, CEVd-infected gynura. Lane 2, non-transformed *A. thaliana.* Lanes 3 to 9, transgenic A. thaliana expressing dt CEVd (+) RNAs with different mutations (#8 to #14, respectively). Lane 10, transgenic A. thaliana expressing dt CEVd (+) RNA wt. *Right panel.* Histograms that represent cleavage of each mutant relative to the wt sequence. (C) *Left panel.* Northern blot analysis of viroid-enriched RNA preparations, the same as in panel (B) *left*, separated by double PAGE*. Right panel.* Histograms that represent ligation of each mutant relative to the wt sequence. Other details as in the legend to Figure 5.

These mutations, besides covering alternative positions of the upper CCR strand, were anticipated as very informative because most impinge on the GAAA tetraloop capping the hairpin that according to Baumstark et al. [25] directs cleavage, and also because most of these nucleotides form part of the loop E that presumably mediates ligation [20,27] (Figure 6A).

The double mutant #8 (C92→G and G99→C, the rationale for the second substitution is given below), and the single mutants #9 (A100→U), #10 (A100→C), #11 (A101→C), #12 (A102→U), and #13 (A102→C), had in general a mild effect on cleavage. Excepting mutant #9, in which cleavage was reduced to 34% with respect to wt, cleavage of the others was at least 68%, with mutants #8, and #11 to #13, reaching 88%–92% (Figure 6B). Given that the GAAA tetraloop belongs to the family of GNRA tetraloops (in which N is any base and R a purine) [37], these results do not support a role in cleavage of the GAAA-capped hairpin, because changes disrupting interactions critical for the tetraloop stability (mutants #8, and #11 to #13) had essentially no influence on cleavage. In contrast, the six mutants induced a pronounced negative effect on ligation, thus sustaining a critical function of loop E in this reaction (Figure 6C). Indeed, from the structural model derived for loop E of PSTVd [27], and considering that nucleotides critical for this motif are conserved or substituted by others preserving it in CEVd, mutants #9, #10, #12, and #13 all introduce non-isosteric pairs disrupting the loop E structure. However, mutant #11 is predicted to maintain the loop E structure, suggesting that its negative effect on ligation could result from sequence rather than from structural restrictions. Consistent with this view, the nucleotide corresponding to position A101 in CEVd is phylogenetically conserved in the family Pospiviroidae (Figure 3).

On the other hand, mutants #11 to #13 affect minimally the stem stability of hairpin I (particularly of its upper portion because they map outside the 3-bp stem adjacent to the tetraloop) and the double-stranded structure (in which they are outside the GC-rich central region of 10 bp containing the cleavage sites) and, therefore, their effects are consistent with a function of this latter structural motif in cleavage (Figure 6A). The negative effects of mutants #9 and #10 on cleavage are also compatible with this view, because they alter the stability of both the 3-bp stem adjacent to the hairpin I tetraloop and the 10-bp central region of the double-stranded structure, although it is difficult to interpret why cleavage was significantly more reduced in mutant #9 than in #10 (Figure 6A and 6B).

Going back to the double mutant #8, its high cleavage (Figure 6B) can be explained because, despite affecting nucleotides C92 and G99 that form a pair phylogenetically conserved in the hairpin I/double-stranded structure of the family Pospiviroidae, this base pair is just inverted (Figure 6A). In mutant #14 (C92→U), in which the pair between C92 and G99 was substituted by a U:G pair, cleavage still was relatively significant (63%). The differential effect in ligation of these two mutants is intriguing: ligation was essentially abolished in mutant #8, whereas in mutant #14 was close to 10% with respect to wt (the highest value for any of the mutants of the present study) (Figure 6C). It is worth noting that the double mutant #8 affects the nucleotide of the upper CCR strand that upon UV irradiation becomes cross-linked to U266 of the lower CCR strand (data not shown in [22]) and also disrupts a G:C pair of the flanking bulged-U helix, in contrast to the single mutant #14 in which this base pair is substituted by a G:U pair (Figure 6A). These results again underline that ligation is influenced by nucleotides aside from those conserved in loop E.

### Mutations in the Lower CCR Strand Affecting Loop E Have a Marked Effect on Ligation but Not in Cleavage of Dimeric CEVd (+) RNAs

**Figure 7:**
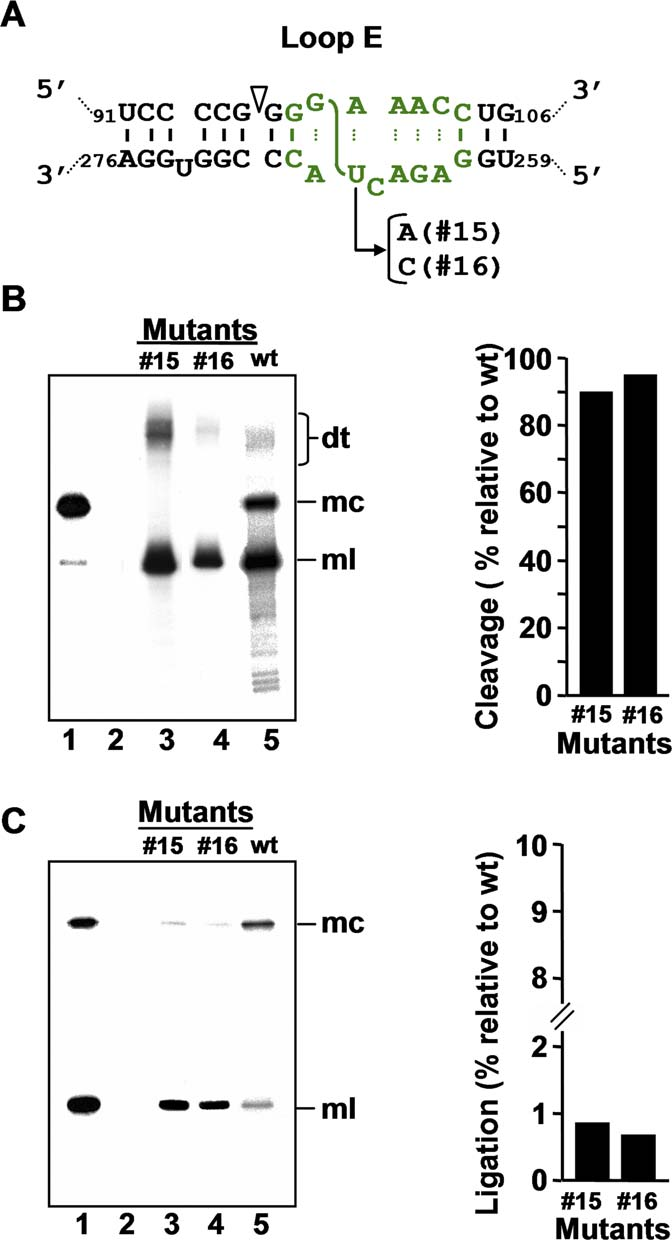
Processing in A. thaliana of Dimeric CEVd Transcripts with Mutations in the Lower CCR Strand Affecting Loop E (A) Rod-like structure, containing the loop E motif and the adjacent bulged-U helix that can form the upper and lower CCR strands. Nucleotide substitutions specific of each mutant (#15 and #16) are indicated. (B) *Left panel.* Northern blot analysis of viroid-enriched RNAs separated by single denaturing PAGE, transferred to a membrane and hybridized with a radioactive riboprobe for detecting CEVd (+) strands. Lane 1, CEVd-infected gynura. Lane 2, non-transformed *A. thaliana.* Lanes 3 and 4, transgenic A. thaliana expressing dt CEVd (+) RNAs with mutations #15 and # 16, respectively. Lane 5, transgenic A. thaliana expressing dt CEVd (+) RNA wt. *Right panel.* Histograms that represent cleavage of each mutant relative to the wt sequence. (C) *Left panel.* Northern blot analysis of viroid-enriched RNAs, the same as in panel (B) *left*, separated by double PAGE*. Right panel.* Histograms that represent ligation of each mutant relative to the wt sequence. Other details as in the legend to Figure 5.

If only nucleotides of the upper CCR strand direct cleavage, its extension should not be influenced by mutations in the lower CCR strand, which in contrast should reduce ligation particularly if they impinge on nucleotides of the loop E motif that presumably directs this reaction. To test this hypothesis, we constructed two mutants in which U266, the nucleotide of the lower CCR strand that upon UV irradiation becomes cross-linked to G99 of the upper CCR strand (data not shown in [22]) (Figure 7A), was changed: mutants #15 (U266→C) and #16 (U266→A). Extending to CEVd the structural model derived for loop E of PSTVd [27], U266 and A100 in loop E of CEVd should interact via *trans* Watson-Crick/Hoogsteen edges and belong to the isosteric subgroup I1. In mutants #15 and #16, C266 and A100, and A266 and A100, are predicted to interact similarly; however, they belong to subgroups I2 and I4, respectively, which are non-isosteric with respect to the original I1 and may thus disrupt the loop E structure to some extent [27]. Northern blot hybridization of RNAs from the corresponding transgenic A. thaliana lines showed that cleavage remained essentially unaffected (90%–95% relative to wt), whereas ligation was essentially abolished (Figure 7B and 7C). These results support further the notion that cleavage is determined exclusively by RNA motifs formed by nucleotides of the upper CCR strand and flanking nucleotides, and also show that ligation is determined by nucleotides of loop E and by others of both CCR strands. In particular, the bulged-U helix may play a key role in aligning the termini to be ligated.

## Discussion

Processing of oligomeric (+) RNAs in the family Pospiviroidae entails cleavage to the ml (+) RNA, and ligation of the resulting species to the mc (+) RNA. Hence, the most direct way to identify the processing site is mapping where the ml (+) RNA intermediate is opened. Previous studies have pointed to the upper strand of the CCR, which, due to its strict conservation within each genera of the family, has been long assumed to play an essential role. Data supporting this view include infectivity bioassays with different PSTVd DNA and RNA constructs [17], with longer-than-unit HSVd clones [16], and with CEVd constructs containing sequence repetitions and point mutations in the upper CCR strand [18]. The latter study concluded that processing occurs at one of three consecutive Gs of the upper CCR strand, and advanced hairpin I or an alternative double-stranded structure as the putative RNA motifs directing cleavage (Figure 5A). A critical reassessment of all these data led to a model involving the double-stranded structure in cleavage, although the model did not predict the mechanism of cleavage-ligation or specify the exact processing site [15]. The infectivity-based approach, however, has an important limitation: bioassays do not provide a linear dose-response, being at best semi-quantitative and making it difficult to draw accurate estimations. Reflecting this limitation, other data point to alternative processing sites in the PSTVd lower CCR strand [28]. Furthermore, transcripts with only a 4-nt repetition of the PSTVd upper CCR strand [38] or with the exact unit-length CEVd [39] are still infectious, and another work suggested that the basic requirement for infectivity of a range of unit-length CEVd in vitro transcripts starting at different domains of the molecule is their ability to form a short double-stranded region of viroid and vector sequences at the junction of the two termini [31]. Therefore, at least in some cases, infectivity is independent of duplicated viroid sequences, possibly because the exact full-length sequence is restored by strand switching of a jumping polymerase during transcription [31]. On the other hand, primer-extension on the ml viroid (+) RNAs isolated from infected propagation hosts also has significant constraints (see Results), with this approach having mapped several processing sites in different PSTVd domains [29,30,32]. Finally, conclusions from in vitro assays in which a potato nuclear extract was primed with an ml PSTVd (+) RNA with a 17-nt repeat of the upper CCR strand should be interpreted with caution, because the processing complex formed in vitro may not mimic the corresponding complex in vivo*.* Moreover, prior to incubation with the nuclear extract, the PSTVd RNA was heated to promote the adoption of a specific secondary structure that may not parallel that existing in vivo [20].

The A. thaliana–based system reported recently [34] circumvents most of these limitations. It is an in vivo system in which the available data indicate that processing is correct: transgenically expressed dt (+) RNAs of typical members of the family Pospiviroidae are cleaved to the ml forms—implying recognition of two identical sites—and then ligated to the infectious mc RNAs, whereas the complementary dt (−) RNAs are not ([34], this work), thus reproducing the situation observed in typical hosts. However, in contrast to typical hosts in which the turnover of the longer-than-unit (+) replicative intermediates is difficult to follow because of their low accumulation and diverse size, the A. thaliana–based system with the viroid-expression cassette integrated in the plant genome provides a constant supply of a size-specific replicative-like intermediate that permits the easy quantification thereof and of its processing products. Moreover, despite typical members of the family Pospiviroidae being able to complete their replication cycle when expressed transgenically as dt (+) RNAs in A. thaliana, the replication level in this non-host plant is rather low (see Figure 1 and [34]), and the ml and mc (+) RNAs can be assumed to come essentially from processing of the transgenically expressed dt (+) RNA. Therefore, the effects of specific mutations in the primary transcript on cleavage and ligation can be evaluated—regardless of whether the resulting products are infectious or not—and it is even possible to identify mutations affecting only ligation.

Our results with the A. thaliana–based in vivo system mapped the cleavage site of CEVd (+) strands at the upper CCR strand, in a position equivalent to that inferred for PSTVd with an in vitro system [20]. However, we consider that the RNA motif directing cleavage in vivo is not the GAAA-capped hairpin proposed previously [20], but the hairpin I/double-stranded structure. The first argument supporting this view is that whereas the cleavage sites of HSVd and ASSVd (+) strands also map at equivalent positions in a similar hairpin I/double-stranded structure, these viroids cannot form the GAAA-capped hairpin. In contrast, examination of the hairpin I/double-stranded structure reveals some appealing features. Hairpin I is composed by a tetraloop, a 3-bp stem, an internal symmetric loop of 1–3 nt in each strand that presumably interact by non-Watson-Crick base pairs [40], and a 9–10-bp stem that can be interrupted by a 1-nt symmetric or asymmetric internal loop [18,35] (Figure 3). Remarkably, these structural features are conserved in the type species of the five genera composing the family Pospiviroidae and additionally: i) the capping tetraloop is palindromic itself, and ii) the two central positions of the tetraloop and the central base pair of the 3-bp stem are phylogenetically conserved (Figure 3) [35]. As a consequence, a long double-stranded structure with a GC-rich central region of 10 bp containing the cleavage sites can be alternatively assumed by the same sequences in a di- or oligomeric RNA (Figure 5A). The second argument supporting the hairpin I/double-stranded structure as the RNA motif directing cleavage derives from the effects on this reaction of mutants affecting differentially this motif versus the GAAA-capped hairpin**.** Chief among them are mutants #8, and #11 to #13 that, despite disrupting interactions crucial for the stability of the GAAA tetraloop, did not basically modify cleavage. Furthermore, because the ml PSTVd (+) RNA with a 17-nt repeat of the upper CCR strand that was used to prime the potato nuclear extract [20] can also form a fragment of the proposed double-stranded structure containing the cleavage sites, the correct cleavage observed in vitro can be alternatively interpreted as being directed by this structure. Our interpretation of direct effects of the introduced mutations in viroid RNA processing is based on the weak viroid RNA-RNA amplification in A. thaliana and, therefore, side effects of this amplification in cleavage and ligation cannot be totally discarded.

**Figure 8:**
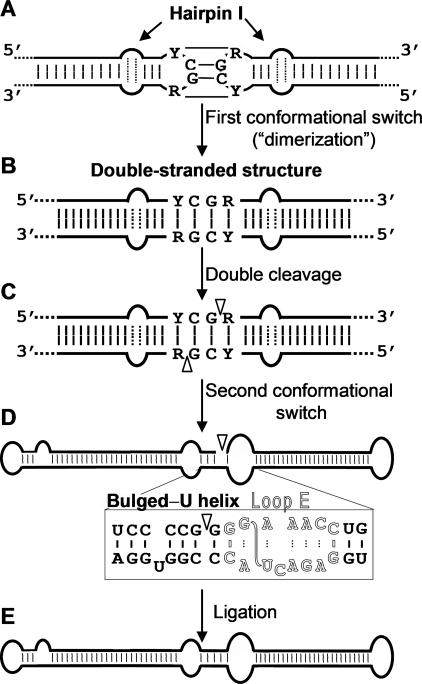
Model for Processing In Vivo of the Oligomeric (+) Replicative Intermediates of the Family Pospiviroidae The model envisages a kissing loop interaction between the palindromic tetraloops of two consecutive hairpin I motifs (A), with their stems forming subsequently a longer interstrand duplex (B). This double-stranded structure is the substrate for cleavage at specific positions in both strands (C). Following a second conformational switch the resulting unit-length strands adopt the extended rod-like structure with loop E (in outlined fonts) and the adjacent bulged-U helix (D), which is the substrate for ligation (E). R and Y refer to purines and pyrimidines, respectively, the S-shaped line denotes the UV-induced cross-link, and white arrowheads mark the cleavage sites in the double-stranded structure and the ligation site in the extended conformation.

In summary, we believe that the substrate for cleavage in vivo of all members of the family Pospiviroidae is the double-stranded structure proposed by Diener [15], with hairpin I playing a role in promoting the adoption of this structure (see below). Although its existence in vivo remains to be fully demonstrated, we have noticed that the cleavage sites in the double-stranded structure leave two 3′-protruding nucleotides in each strand (Figure 8), the characteristic signature of RNase III enzymes [41,42]. The participation of an enzyme of this class, of which there are at least seven in A. thaliana [43], is consistent with the nuclear location of some of them, which additionally have preference for substrates with a strong secondary structure resembling that of viroids. Moreover, one or more RNase III isozymes should be involved in the genesis of the viroid-derived small RNAs with properties similar to the small interfering RNAs that accumulate in viroid-infected tissues [44–48]. Going one step further, if an RNase III indeed catalyzes cleavage of the oligomeric (+) RNAs of the family Pospiviroidae, the resulting products should have 5′-phosphomonoester and free 3′-hydroxyl termini. Characterization of the ml (+) RNAs from A. thaliana transgenically expressing dt CEVd (+) RNAs shows that this is actually the case (M. E. Gas, D. Molina-Serrano, C. Hernández, R. Flores, and J. Daròs, unpublished data). The adoption in vivo of the double-stranded structure with a GC-rich central region containing the cleavage sites could be promoted by hairpin I because prior work with PSTVd has mapped a dimerization domain at this hairpin [40]. This situation resembles that observed previously in retroviruses in which dimerization, a critical step of their infectious cycle, is mediated by a hairpin with a palindromic loop that can dimerize co- or post-transcriptionally via a kissing loop interaction between two viral RNAs [49]. During transcription of oligomeric (+) RNAs of the family Pospiviroidae, a kissing loop interaction between the palindromic tetraloops of two consecutive hairpin I motifs might similarly start intramolecular dimerization, with their stems then forming a longer interstrand duplex (Figure 8). Part of the negative effects on cleavage of mutants #1 to #6 (Figure 5) could result from weakening dimerization.

Regarding ligation, our results support that the substrate for this reaction in the genus *Pospiviroid* is the extended conformation containing loop E [20,22] (Figure 8)*.* Therefore, whereas cleavage is solely dependent on the upper CCR strand and flanking nucleotides, ligation is dependent on nucleotides of both CCR strands that encompass those of loop E and others adjacent. This entails a switch between two conformations, one for cleavage and another for ligation, which might be facilitated by the RNA helicase activity associated with some RNase III enzymes [43]. Because within the family Pospiviroidae loop E is only formed in the genera *Pospiviroid* and *Cocadviroid*, other genera of this family must have alternative motifs playing a similar role in ligation. Potential candidates are the extended conformation of the CCR with a bulged-U helix conserved in all members of the genera *Pospiviroid*, *Hostuviroid* and *Cocadviroid*, and similar structures in the other genera of this family. Last but not least, the 5′-phosphomonoester and free 3′-hydroxyl termini resulting from cleavage mediated by an RNase III predict the existence of an RNA ligase able to join these ends, which is therefore different from the class represented by the wheat-germ RNA ligase that catalyzes joining between 5′-hydroxyl and 2′,3′-cyclic phosphodiester termini [50]. This latter RNA ligase class has been long regarded as the enzyme involved in circularization of PSTVd (+) strands and, by extension, of other members of its family [29,51]. Our results advise for a reassessment of this long-held paradigm. The A. thaliana–based system is a promising tool for dealing with this and other related questions because it combines the advantages described previously with a broad collection of mutants.

## Materials and Methods

### Viroids and plasmid constructs.

Viroid sequence variants were CEVd (M34917) having a deleted G between positions 70 and 74, HSVd (Y09352), and ASSVd (AF421195). Plasmids pBmCEVdB, pBmCEVdS, and pBmCEVdP contained monomeric CEVd-cDNAs cloned at the BamHI, SacI, and PstI sites, respectively, pBmHSVdE a monomeric HSVd-cDNA cloned at the EcoRI site, and pBmASSVdS a monomeric ASSVd-cDNA cloned at the SalI site. Plasmids containing head-to-tail dimeric cDNA inserts of CEVd, HSVd, and ASSVd have been described previously [34]. Binary vectors for plant transformation were constructed by replacing the β-glucuronidase-cDNA of pCAMBIA-2301 (AF234316) by dimeric head-to-tail CEVd-cDNAs (starting at the PstI site) corresponding to the wt (pCKdCEVd-wt) and 16 mutants (pCKdCEVd-1 to pCKdCEVd-16) (Table 1).

### Site-directed mutagenesis.

Plasmid pBmCEVdP was amplified with a series of pairs of 5′-phosphorylated adjacent primers that were complementary and homologous to different regions of the wt CEVd sequence, except in some 5′-proximal positions in which changes were introduced to obtain the desired mutants (Table 1). *Pwo* DNA polymerase was used in the buffer recommended by the supplier (Roche Applied Science). After initial heating at 94 °C for 2 min, the amplification profile (30 cycles) was 30 s at 94 °C, 30 s at 58–68 °C (depending on the predicted melting temperatures), and 3.5 min at 72 °C, with a final extension of 10 min at 72 °C. PCR products corresponding to the full-length plasmid were eluted after agarose gel electrophoresis, ligated, and used for transformation. Incorporation of the expected mutations was confirmed by sequencing. The mutated CEVd-cDNA inserts were PCR-amplified, eluted after non-denaturing PAGE, and ligated to obtain dimeric cDNAs that were cloned in pBluescript II KS (+). Plasmids with dimeric head-to-tail inserts were selected by restriction analysis and subcloned in the binary vector pCAMBIA-2301.

### Transgenic plants.

Agrobacterium tumefaciens (strain C58C1) was transformed with plasmids (pCKdCEVd-wt and pCKdCEVd-1 to pCKdCEVd-16) following standard protocols. Transformation of A. thaliana (ecotype Col-0) was performed by the floral dip method using midlog-grown cultures of *A. tumefaciens* [52], and transgenic plants were selected by germinating the seeds from dipped A. thaliana in plates with 100 μg/ml kanamicine, 300 μg/ml cefotaxime, and 10 μg/ml benomyl.

### RNA analysis.

Total nucleic acids from leaves of CEVd-infected gynura (Gynura aurantiaca DC), HSVd-infected cucumber (Cucumis sativus L.), and transgenic A. thaliana, as well as from fruits of ASSVd-infected apple (Malus pumilla Mill.), were extracted with buffer-saturated phenol and enriched in viroid RNAs by chromatography on non-ionic cellulose (CF11, Whatman) [34]. RNAs from CEVd-infected gynura and ASSVd-infected apple were further fractionated with 2 M LiCl.

RNA aliquots were separated by either single denaturing PAGE in 5% gels with 8 M urea in 1X TBE (89 mM Tris, 89 mM boric acid, 2.5 mM EDTA [pH 8.3]), or double PAGE, first in a non-denaturing 5% gel in TAE (40 mM Tris, 20 mM sodium acetate, 1 mM EDTA [pH 7.2]), with the gel segment containing the monomeric viroid RNAs being cut and applied on top of a second 5% gel with 8 M urea in 0.25X TBE. RNAs were electroblotted to nylon membranes (Hybond-N, Amersham Biosciences), UV-fixed with a cross-linker (Hoefer), and hybridized (at 70 °C in the presence of 50% formamide) with strand-specific ^32^P-labeled riboprobes obtained by transcription with T3 or T7 RNA polymerases of plasmid pBdCEVdP properly linearized. After washing the membranes, the signals of the dt RNAs and their resulting ml and mc forms were quantified with a bioimage analyzer (Fujifilm FLA-5100). Cleavage and ligation were estimated for each A. thaliana CEVd mutant from the fractions (mc+ml)/(dt+mc+ml) and mc/(mc+ml), respectively, and the results normalized with respect to those of the A. thaliana CEVd-wt (taken as 100%). Two independent plants were analyzed for each transgenic line, with differences in cleavage and ligation being less than 10% in all instances.

Primer extensions were carried out for 45 min at 55 °C, 10 min at 60 °C, and 5 min at 65 °C, in 20 μl containing 50 mM Tris-HCl [pH 8.3], 75 mM KCl, 3 mM MgCl_2_, 5 mM dithiothreitol, 0.5 mM each of the dNTPs, 40 U of RNase inhibitor (Promega), and 200 U of SuperScript III reverse transcriptase (Invitrogen). The ml and mc viroid RNAs serving as template were obtained by double PAGE and elution. Primers PI (5′-TTCTCCGCTGGACGCCAGTGATCCGC-3′), PII (5′-GCTTCAGCGACGATCGGATGTGGAGCC-3′), PIII (5′- GAGCAGGGGTGCCACCGGTCGC-3′), and PIV (5′-GACTAGCGGCGCGAAGAGTAGGTGG-3′), were 5′-labeled with T4 polynucleotide kinase (Roche Applied Science) and [γ-^32^P]ATP (Amersham Biosciences). Before reverse transcription, each primer was annealed in water to the purified viroid RNA (10:1 molar ratio) by heating at 95 °C for 5 min and snap-cooling on ice. Reactions were stopped at 70 °C for 15 min, and the products analyzed by PAGE on 6% sequencing gels. The exact size of the extension products was determined by running in parallel sequence ladders obtained with the corresponding primer and a recombinant plasmid containing the monomeric viroid-cDNA insert (Thermo Sequenase cycle sequencing kit, USB).

## Supporting Information

Figure S1 Alternative RNA Motifs Formed by the Upper CCR Strand and Flanking Nucleotides Proposed to Direct Cleavage of the Oligomeric (+) Strands of the Family Pospiviroidae Following cleavage, a conformational switch occurs leading to the loop E–containing rod-like structure that promotes ligation. Blue and red lines indicate nucleotides of the upper and lower CCR strands, respectively.
(1.1 MB TIF)

Figure S2 Primer Extensions on the Monomeric Linear CEVd RNA from a Transgenic A. thaliana Line Expressing the Dimeric CEVd (+) RNA (A) The cDNAs generated with the complementary primers PI, PIV, and PII were separated by denaturing PAGE (lanes 1 to 3, respectively) in parallel with DNA markers with their size in nucleotides indicated on the right. Predominant cDNAs are denoted by asterisks. (B) Rod-like secondary structure predicted for CEVd, with the upper and lower insets highlighting two portions thereof. Positions of the complementary primers PI, PIV, and PV are indicated with arrows and bold fonts.
(1.6 MB TIF)

## Abbreviations

ASSVd: apple scar skin viroid
CbVd-1: coleus blumei viroid 1
CCR: central conserved region
CEVd: citrus exocortis viroid
dt: dimeric transcript
HSVd: hop stunt viroid
mc: monomeric circular
ml: monomeric linear
PSTVd: potato spindle tuber viroid
wt: wild-type
