# Supplementary figures and images for: Processing of Nuclear Viroids In Vivo: An Interplay between RNA Conformations
Source: PLoS Pathog. 2007 Nov 30;3(11):e182. doi: 10.1371/journal.ppat.0030182 (PMC2098832; doi:10.1371/journal.ppat.0030182)

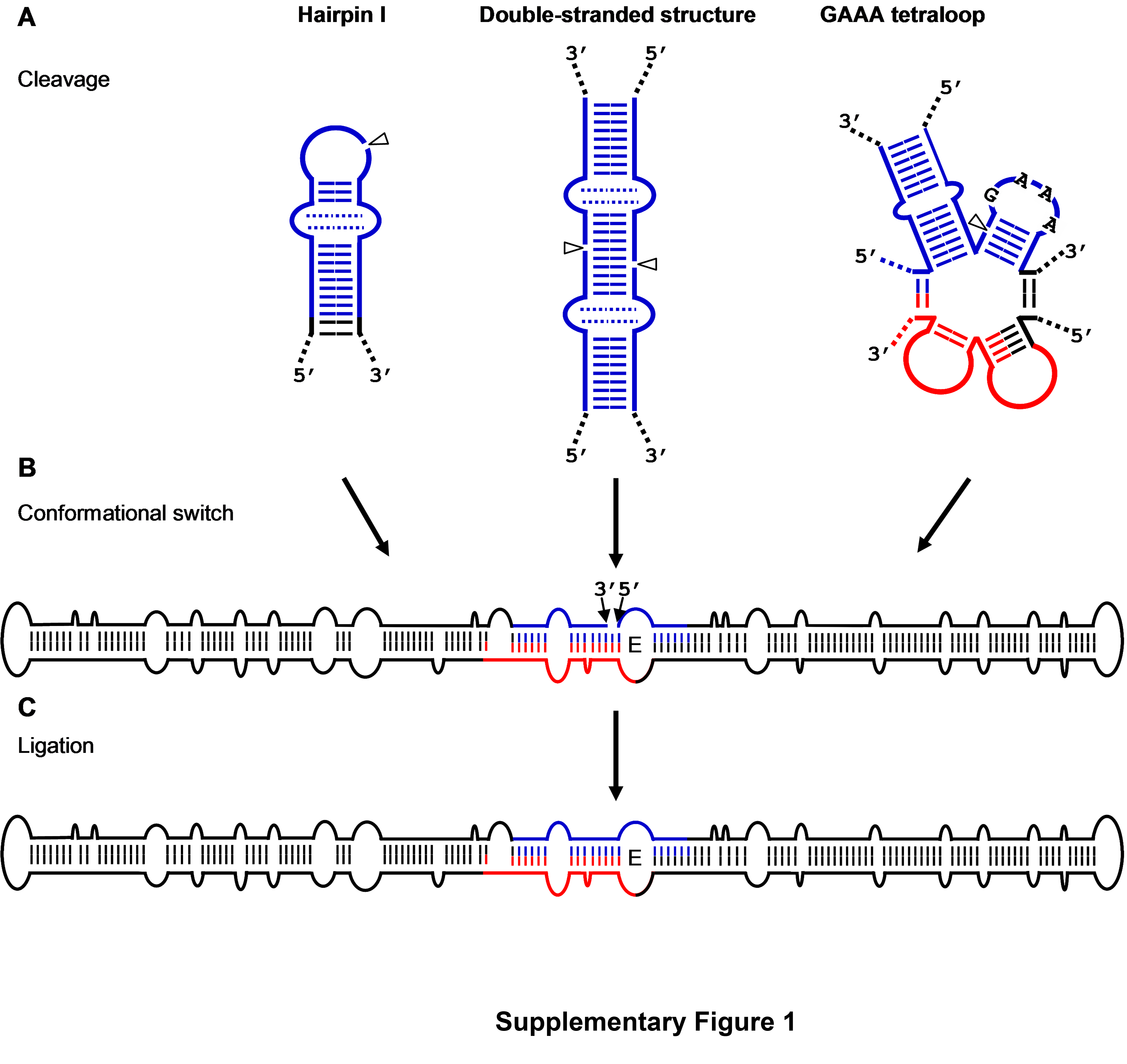

Supplement: Figure S1 — Following cleavage, a conformational switch occurs leading to the loop E–containing rod-like structure that promotes ligation. Blue and red lines indicate nucleotides of the upper and lower CCR strands, respectively. (1.1 MB TIF) [file ppat.0030182.sg001.tif]

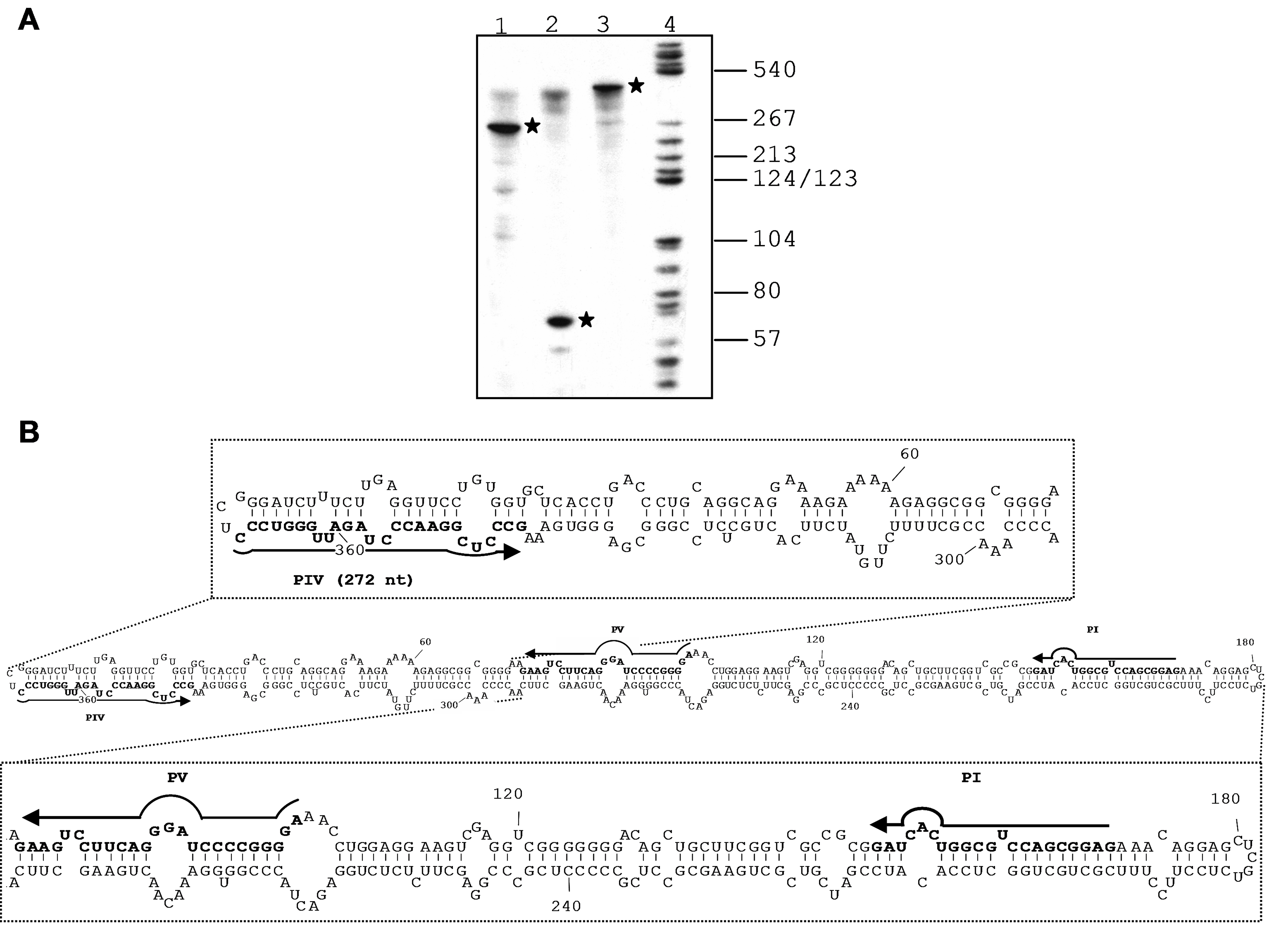

Supplement: Figure S2 — (A) The cDNAs generated with the complementary primers PI, PIV, and PII were separated by denaturing PAGE (lanes 1 to 3, respectively) in parallel with DNA markers with their size in nucleotides indicated on the right. Predominant cDNAs are denoted by asterisks. (B) Rod-like secondary structure predicted for CEVd, with the upper and lower insets highlighting two portions thereof. Positions of the complementary primers PI, PIV, and PV are indicated with arrows and bold fonts. (1.6 MB TIF) [file ppat.0030182.sg002.tif]
